# Safety and efficacy of COVID-19 vaccine immunization during pregnancy in 1024 pregnant women infected with the SARS-CoV-2 Omicron virus in Shanghai, China

**DOI:** 10.3389/fimmu.2023.1303058

**Published:** 2024-01-16

**Authors:** Hongmei Deng, Yinpeng Jin, Minmin Sheng, Min Liu, Jie Shen, Wei Qian, Gang Zou, Yixin Liao, Tiefu Liu, Yun Ling, Xiaohong Fan

**Affiliations:** ^1^ Department of Gynecology and Obstetrics, Shanghai Public Health Clinical Center, Fudan University, Shanghai, China; ^2^ Liver Disease Center, Shanghai Public Health Clinical Center, Fudan University, Shanghai, China; ^3^ Obstetrics and Gynecology Hospital of Fudan University, Shanghai, China; ^4^ International Peace Maternity & Child Health Hospital Affiliated to Jiaotong University, Shanghai, China; ^5^ Department of Fetal Medicine & Prenatal Diagnosis Center, Shanghai First Maternity and Infant Hospital, School of Medicine, Tongji University, Shanghai, China; ^6^ Scientific Research Center, Shanghai Public Health Clinical Center, Shanghai, China; ^7^ Department of Infectious Disease, Shanghai Public Health Clinical Center, Fudan University, Shanghai, China; ^8^ Department of Respiratory, Shanghai Public Health Clinical Center, Fudan University, Shanghai, China

**Keywords:** COVID-19 vaccination, Omicron strain infection, pregnancy, newborns, breastfeeding, vertical transmission

## Abstract

**Background:**

Large sample of pregnant women vaccinated with COVID-19 vaccine has not been carried out in China. The objective of this study was to evaluate the safety and effectiveness of COVID-19 inactivated vaccine in pregnant women infected with the SARS-CoV-2 Omicron variant.

**Methods:**

A total of 1,024 pregnant women and 120 newborns were enrolled in this study. 707 pregnant women received one to three doses of the inactivated COVID-19 vaccine, and 317 unvaccinated patients served as the control group. A comparison was made between their clinical and laboratory data at different stages of pregnancy.

**Results:**

The incidence rate of patients infected with Omicron variant in the first, the second, and the third trimesters of pregnancy was 27.5%, 27.0%, and 45.5% in patients during, respectively. The corresponding length of hospital stay was 8.7 ± 3.3 days, 9.5 ± 3.3 days, and 11 ± 4.3 days, respectively. The hospitalization time of pregnant women who received 3 doses of vaccine was (8.8 ± 3.3) days, which was significantly shorter than that of non-vaccinated women (11.0 ± 3.9) days. (P<0.0001). The positive rate of SARS-CoV-2 IgG in patients in the early stage of pregnancy was 28.8%, while that in patients in the late stage of pregnancy was 10.3%. However, three-doses of vaccination significantly increased the SARS-CoV-2 IgG positive rate to 49.5%. The hospitalization time of SARS-CoV-2 IgG-positive patients was shorter than that of negative patients (9.9 ± 3.5 days), which was 7.4 ± 2.0 days. 12.2% of vaccinated women experienced mild adverse reactions, manifested as fatigue (10.6%) and loss of appetite (1.6%). The vaccination of mother did not affect her choice of future delivery mode and the Apgar score of their newborn. All newborns tested negative for SARS-CoV-2 nucleic acid, as well as for IgG and IgM antibodies.

**Conclusions:**

Women in the third trimester of pregnancy are highly susceptible to infection with the Omicron strain. The vaccination of pregnant women with COVID-19 vaccine can accelerate the process of eliminating SARS-CoV-2 virus, and is considered safe for newborns. The recommended vaccination includes three doses.

## Introduction

Since the 2019 coronavirus disease (COVID-19) caused by severe acute respiratory syndrome coronavirus type 2 (SARS-CoV-2) infection was first announced in January 2020, the number of confirmed cases and their related incidence rate and mortality have increased rapidly (1). According to the COVID-19 Excess Mortality Collaborators’ modeling, from January 1, 2020 to December 31, 2021, about 18 million people around the world have died of COVID-19, which is three times the official report (2). Since the emergence of the D614G variant, SARS-CoV-2 has continued to mutate, resulting in the development of various mutated strains. The World Health Organization (WHO) declared these variants as Variant of Concern (VOC) due to their increased public threat. Among them, Delta and Omicron strains are the most concerned. In particular, the Omicron variant has emerged as a global epidemic virus and exhibits higher infectivity than other variants, but with less virulence (3). As of July 2, 2022, the Omicron variant has caused 649,657 infections in Shanghai, China (4). The phylogenetic characteristics of the SARS-CoV-2 genomes from 129 patients confirmed the current Omicron BA.2.2 sub-lineage pandemic in Shanghai (5).

Pregnant women have always been considered a high-risk group, as they are particularly susceptible to respiratory pathogens and severe pneumonia during pregnancy due to the physiological and immunological changes, such as diaphragm elevation, increased oxygen consumption, and airway mucosal edema. For example, the 1918 influenza pandemic was the most serious pandemic before COVID-19, with a total mortality rate of 2.6% and a maternal mortality rate of 37% (6). Similarly, in 2003, the SARS pandemic led to a mortality rate of 25%, and about 50% of infected pregnant women were treated in the intensive care units (ICUs), and 33% required mechanical ventilation (7). In contrast, pregnant women with COVID-19 were three times more likely to need ICU care, 2.4 times more likely to need extracorporeal membrane oxygenation (ECMO), and 1.7 times more likely to die (8). Therefore, pregnancy serves as a risk factor for severe COVID-19.

Studies have confirmed that vaccination of COVID-19 vaccine can reduce the risk of severe illness caused by COVID-19. At present, the approved vaccines of COVID-19 include mRNA vaccine, adenovirus vector vaccine, and inactivated vaccine. Inactivated vaccines, also known as whole-virus vaccines, have lost their pathogenic ability but retain their immunogenicity. Due to their relative stability and safety, they have become the most frequently used COVID-19 vaccine in China. However, data on the protective effect of COVID-19 vaccine are mainly based on the general population. The vaccination rate of pregnant women is significantly lower than that of non-pregnant women of childbearing age, and no large-scale population studies have been conducted on women who are conceiving, pregnant, or breastfeeding (9). As such, the safety and effectiveness of COVID-19 vaccine for pregnant and lactating women and their offspring are still uncertain.

The purpose of this study was to evaluate the safety and efficacy of inactivated COVID-19 vaccines in a sample of pregnant women infected with the Omicron strain of COVID-19, as well as their newborns. We discovered that patients who received vaccination had rapid clearance of the virus, mild clinical symptoms, and short hospitalization time. In addition, their newborns tested negative for COVID-19. Only a small proportion experienced mild adverse reactions to vaccination. Therefore, we conclude that inactivated vaccines are safe and effective for pregnant women and their newborns, and it is recommended to follow the three-dose vaccination regimen as it is more effective.

## Materials and methods

### Patients

We conducted a retrospective cohort study of a total of 1024 pregnant women diagnosed with COVID-19 Omicron strain infection by the Shanghai Center for Disease Control and Prevention and hospitalized at the Shanghai Public Health Clinical Center in Shanghai, China from February 28, 2022 to May 3, 2022. All pregnant women included in this study were infected with COVID-19 Omicron strain for the first time due to the zero clearing policy implemented in Shanghai. Most pregnant women in this study were vaccinated first and infected with COVID-19 later. This study was conducted in accordance with the protocol (2022-S067-01) approved by the Ethics Committee of the Shanghai Public Health Clinical Center. According to the Diagnosis and Treatment Plan for Novel Coronavirus Pneumonia (Trial Ninth Edition), the patient’s epidemiological history, clinical manifestations, and laboratory tests were comprehensively evaluated and diagnosed. COVID-19 infection was confirmed by positive results of nucleic acid test. Other patients with lung infections were excluded from the study.

Of the 1,024 patients, 282 were in the early stage of pregnancy, 276 were in the second trimester, and 466 were in the third trimester. Among all cases, 865 were asymptomatic, 157 had mild symptoms, two patients had moderate symptoms, and no patients had severe or critical symptoms. Because of the zero clearing policy implemented in Shanghai, the discharge standard of patients is that COVID-19 nucleic acid turns negative. Patients were discharged if they met all four criteria (1): body temperature remains normal for more than 3 days (2), respiratory symptoms significantly improve (3), lung imaging shows significant improvement in acute exudative lesions, and (4) respiratory specimens have two consecutive negative viral nucleic acid tests (sampling time interval of at least 24 hours) and PCR computed tomography (CT) values of N and ORF genes are ≥35.

A total of 707 patients received one to three doses of inactivated COVID-19 vaccine. The manufacturers of vaccines received by our study cohort include China’s Biological Products Research Institute Co., Ltd., Wuhan Institute of Biological Products Co., Ltd., and Sinovac Biotech Co., Ltd. According to the information provided by the three manufacturers, their production of vaccines proceeded with similar procedures, including the usage of African green monkey kidney Vero cells for virus amplification, the subsequent inactivation step subjecting viruses to effective inactivation while retaining the immunogenicity of the antigen components, and the addition of adjuvant-aluminum hydroxide to boost the immunogenicity of the vaccine. Given that our study subjects received the same type of vaccine produced following a similar manufacturing process, we did not distinguish them according to their manufacturers. As a retrospective study, we also did not use the interval between vaccinations as a distinguishing factor. Within the study cohort, the intervals between the second and first shots had an approximate range of 3-6 weeks, and those between the third and second shots were about 6-12 months. We only grouped patients according to the total number of SARS-CoV-2 vaccines they received. Of the 707 vaccinated patients, 685 were vaccinated before becoming pregnancy, and 22 were vaccinated without knowing they were pregnant. According to the vaccination record, the patients were divided into four groups: unvaccinated group (n = 317), one-dose vaccine group (n = 56), two-dose vaccine group (n = 546), and three-dose vaccine group (n = 105). The time of sample collection was the first blood draw and nucleic acid test during the patient’s admission to the hospital.

### Data analysis

**Figure 1:**
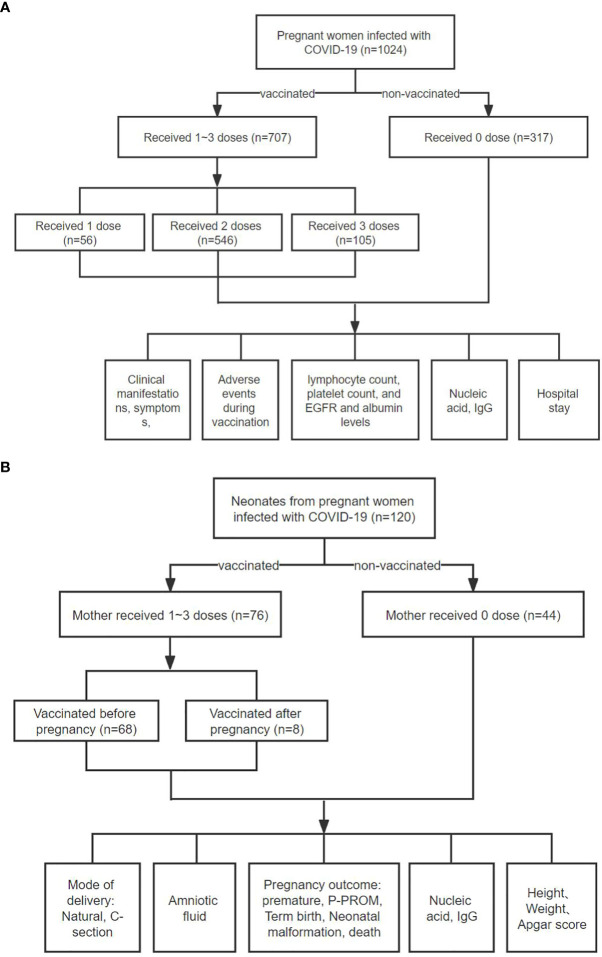
Flowchart of this study. **(A)** A total of 1,024 pregnant women diagnosed with COVID-19 Omicro infection were selected. According to the number of doses administered, patients were divided into four groups: unvaccinated group (n = 317), one dose vaccine group (n = 56), two-dose vaccine group (n = 546), and three-dose vaccine group (n = 105). Subsequently, we compared various clinical indicators of these groups of pregnant women. **(B)** A total of 118 COVID-19 patients gave birth to 120 newborns during hospitalization. Among them, 76 patients were vaccinated with inactivated COVID-19 vaccine, and 42 patients were not vaccinated. Among 76 vaccinated patients, 68 were vaccinated before pregnancy and 8 were vaccinated during pregnancy. Subsequently, we compared the pregnancy and neonatal outcomes between the two groups of patients, such as mode of delivery, newborn’s weight and height, etc. Here, the term “Mother” is conveniently used throughout the flow chart, although at the initial vaccination, the subjects were either mothers-to-be or not yet pregnant.

All pregnant women infected with the SARS-CoV-2 Omicron strain received standard treatment according to the guidelines provided by the World Health Organization (WHO). Clinical data, including symptoms, chest CT images, and laboratory test results, were collected and compared, as follows: (1) clinical presentations at admission, SARS-CoV-2 nucleic acid PCR CT value, SARS-CoV-2-specific IgG and IgM antibody levels, and other blood biochemical indicators such as transaminase, creatinine, etc. (2) clinical presentation of Omicron-infected patients at different stages of pregnancy; (3) length of hospitalization. In order to determine whether maternal vaccination affects newborns, and whether Omicron infection is transmitted through vertically transmission or breastfeeding, we will analyze the clinical characteristics of their newborns. A multiple linear regression analysis was conducted to examine factors that may affect maternal hospitalization time. (**Figures 1A, B**).

### Statistics

The statistical analysis was carried out using the SPSS 25.0 software. The skewed distribution of the measurement data was denoted as M (Q1, Q3). The Mann–Whitney U test was utilized to analyze inter-group comparisons of continuous numerical variables with non-normal distribution. Counting data was expressed as the number of examples (percentages). The categorical variables were compared between groups using the χ2 test or the Fisher exact probability method. The Spearman correlation test was used to analyze the correlation between the length of hospital stay and various influencing factors. Multifactor analysis was carried out through the use of multivariate linear regression analysis. The survival curve was plotted using GraphPad 9.3.1. All the tests were conducted on both sides. P<0.05 was considered statistically significant.

## Results

### Clinical manifestations of pregnant women infected with the Omicron variant of SARS-CoV-2

The 1024 pregnant women infected with the Omicron BA.2.2 subline virus were aged between 18 and 46 years, with a median age of 30 years (range: 27-33 years). (**Table 1**) Among them, 82.9% were in the normal pregnancy age range (18–34 years), while 17.1% were in the advanced maternal age group (≥35 years). The main symptoms at admission were fever (3.7%), cough and shortness of breath (6.1%), vomiting (0.8%), diarrhea (0.2%), and rash (4.9%), while the remaining (84.6%) were asymptomatic. The clinical manifestations were not affected by vaccination or vaccination dose (P>0.05) (**Table 1**).

**Table 1:** Clinical characteristics of COVID-19 in pregnant women.

Subject (case, %)	Total (n = 1,024)	Doses of vaccine					Statistical value	*P*
		(A) 0 dose (n = 317)		(B) 1 dose (n = 56)	(C) 2 doses (n = 546)	(D) 3 doses (n = 105)		
Age (years)^#^							χ^2 = ^3.831	0.2803
18–34	849 (82.9)	255 (80.4)		46 (82.1)	455 (83.3)	93 (88.6)		
≥35	175 (17.1)	62 (19.6)		10 (17.9)	91 (16.7)	12 (11.4)		
Pregnancy stage^#^							χ^2 = ^113.4	< 0.0001
Early	282 (27.5)	58 (18.3)		12 (21.4)	146 (26.7)	66 (62.9)	χ^2 = ^80.43	< 0.0001
Middle	276 (27.0)	74 (23.3)		17 (30.4)	150 (27.5)	35 (33.3)	χ^2 = ^4.673	0.1974
Late	466 (45.5)	185 (58.4)		27 (48.2)	250 (45.8)	4 (3.8)	χ^2 = ^94.92	< 0.0001
Pregnancy risk^a^							χ^2 = ^3.149	0.7899
Purple + green	535 (52.2)	164 (51.7)		30 (53.6)	278 (50.9)	63 (60.0)	χ^2 = ^2.990	0.3931
Purple + yellow	379 (37.0)	117 (36.9)		20 (35.7)	209 (38.3)	33 (31.4)	χ^2 = ^1.822	0.6102
Purple + orange	110 (10.7)	36 (11.4)		6 (10.7)	59 (10.8)	9 (8.6)	χ^2 = ^0.643	0.8865
Clinical manifestations^#^								
Asymptomatic	865 (84.5)		263 (83.0)	49 (87.5)	461 (84.4)	92 (87.6)	χ^2 = ^0.973	0.6147
Mild^b^	157 (15.3)		54 (17.0)	7 (12.5)	83 (15.2)	13 (12.4)	χ^2 = ^0.772	0.6798
Moderate^b^	2 (0.2)		0 (0)	0 (0)	2 (0.2)	0 (0)	–	>0.9999
Clinical symptoms^#^								
Fever	38 (3.7)	14 (4.4)		0 (0)	21 (3.8)	3 (2.9)	–	0.4747
Coughing/difficulty breathing	62 (6.1)	21 (6.6)		1 (1.8)	33 (6.0)	7 (6.7)	–	0.6708
Vomiting	8 (0.8)	3 (0.9)		0 (0)	5 (0.9)	0 (0)	–	0.7085
Diarrhea	2 (0.2)	1 (0.3)		0 (0)	1 (0.2)	0 (0)	–	0.5235
Rashes	5 (4.9)	3 (0.9)		0 (0)	1 (0.2)	1 (0.9)	–	0.1755
**Image changes^#^**	2 (0.2)	0 (0)		0 (0)	2 (0.2)	0 (0)	–	>0.9999

^#^The data were collected upon admission.

-Comparison between the vaccinated and unvaccinated groups

a The National Health and Family Planning Commission has released a female pregnancy risk assessment table, which is divided into five colors according to the severity of the risk: green (low risk), yellow (general risk), orange (high-risk), red (extremely high-risk), and purple (infectious disease).

b The definition of “mild” was stated as mild clinical symptoms without any alterations in the computed tomography (CT) images; The definition of “moderate” was assigned as mild clinical symptoms accompanied by alterations in CT images.

A total of 466 patients, accounting for 45.5% of the patients, were in the third trimester of pregnancy, which was significantly more than the first and second trimesters, with 282 cases (27.5%) and 276 cases (27.0%), respectively. (**Supplementary Table S1**). About 13.5% of patients in the third trimester of pregnancy had high-risk factors (orange/high-risk), which was significantly higher than the first and second trimester, with 7.1% and 9.8%, respectively (P = 0.0190). The hospitalization time for the third trimester population was 11 ± 4.3 days, which was significantly longer than the first trimester population’s 8.7 ± 3.3 days (P<0.0001) and the second trimester population’s 9.5 ± 3.3 days (P = 0.0004). (**Supplementary Table S1**).

### The effect of vaccine dose on COVID-19 in pregnant women

Among 1,024 patients, 707 (69.0%) have received 1-3 doses of inactivated COVID-19 vaccine. Their interval between vaccinations were not fixed. The interval between the second and first shots was about 3-6 weeks, and the interval between the third and second shots was about 6-12 months. 12.3% of pregnant women who received the vaccine experienced mild adverse reactions, mainly including fatigue (10.6%), decreased appetite (1.6%), and rash (0.1%). The occurrence of adverse reactions was not related to vaccine dose (P = 0.8065). (**Table 2**).

**Table 2:** The effect of different vaccine doses on pregnant women infected with COVID-19.

Subject (case, %)	Total (n = 1,024)	Doses of vaccine				Statistical value	*P*
		(A) 0 dose (n = 317)	(B) 1 dose (n = 56)	(C) 2 doses (n = 546)	(D) 3 doses (n = 105)		
**Adverse events during vaccination**^a^	179 (12.2)	/	15 (26.8)	140 (25.6)	24 (22.9)	χ^2 = ^0.4302	0.8065
Fatigue	155 (10.6)	/	13 (23.2)	121 (22.2)	21 (20.0)	χ^2 = ^0.2995	0.8609
Poor appetite	23 (1.6)	/	2 (3.6)	18 (3.3)	3 (2.9)	χ^2 = ^0.0736	0.9639
Rashes	1 (0.1)	/	0 (0)	1 (0.2)	0 (0)		
SARS-CoV-2 antibodies^#^							
Positive SARS-COV-2 IgG	180 (18.4)	14 (4.7)	10 (19.2)	106 (20.3)	50 (49.5)	χ^2 = ^104.1	<0.0001
Positive SARS-COV-2 IgM	5 (0.5)	1 (0.3)	0 (0)	2 (0.4)	2 (2.0)	–	>0.9999
**Ct value (N gene)^#^**	29.2 ± 8.4	28.2 ± 8.4	29.8 ± 7.6	29.4 ± 8.4	30.4 ± 8.8	A vs. B	0.1220
						A vs. C	0.0442
						A vs. D	0.0444
**Ct value (ORF1ab)^#^**	31.3 ± 8.6	30.3 ± 8.6	32.3 ± 7.8	31.6 ± 8.5	32.5 ± 8.7	A vs. B	0.0804
						A vs. C	0.0520
						A vs. D	0.0529
**Hospital stay (days)**	9.8 ± 3.8	11.0 ± 3.9	9.8 ± 5.4	9.5 ± 3.7	8.8 ± 3.3	A vs. B	0.0154
						A vs. C	<0.0001
						A vs. D	<0.0001

^#^The data were collected upon admission.

-Comparison between the vaccinated and unvaccinated groups.

a Person-time.

Because of the zero clearing policy implemented in Shanghai, the discharge standard of patients is that COVID-19 nucleic acid turns negative. The average hospitalization time of pregnant women who received three doses of vaccine was 8.8 ± 3.3 days, significantly shorter than 11.0 ± 3.9 days of unvaccinated patients (P<0.0001). This difference was significantly higher than 28.2 ± 8.4 of unvaccinated patients (P=0.0444). (**Table 2**).

Overall, about 10.3% of late pregnancy population tested positive for SARS-CoV-2 IgG, significantly lower than 28.8% of early pregnancy population (P<0.0001). (**Supplementary Table S1**). However, about 49.5% of pregnant women tested positive for SARS-CoV-2 IgG after receiving three doses of vaccine, significantly higher than 4.7% of the unvaccinated group, 19.2% of the one-dose vaccine group, and 20.3% of the two-dose vaccine group (P<0.0001). (**Table 2**). The average PCR Ct value of N gene in pregnant women who received three doses of vaccine at the time of admission was 30.4 ± 8.8. (**Table 2**). The average hospitalization time of pregnant women who received three doses of vaccine was 8.8 ± 3.3 days, significantly shorter than 11.0 ± 3.9 days of unvaccinated patients (P<0.0001). This difference was significantly higher than 28.2 ± 8.4 of unvaccinated patients (P = 0.0444). (**Table 2**).

**Figure 2:**
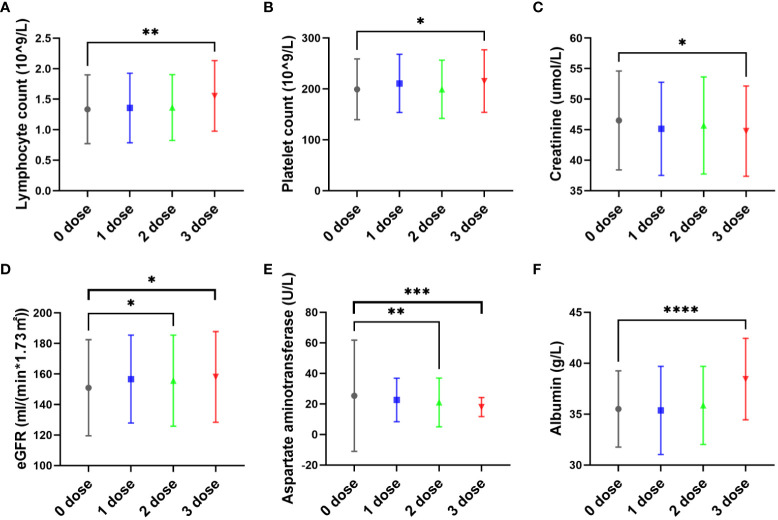
The effect of vaccine dose on pregnant women with COVID-19 infection. The pregnant women in this study had received zero, one, two, or three doses of inactivated SARS-CoV-2 vaccines before or after pregnancy prior to infection with the SARS-CoV-2 Omicron variant. Most pregnant women in this study were vaccinated first and infected with COVID-19 later. At admission, patients who had received three doses of vaccine had higher lymphocyte (Median: 1.575*10^9/L) compared to patients who had not been vaccinated (Median: 1.300*10^9/L) (P<0.05) **(A)**, higher platelet counts (Median: 210.5*10^9/L) compared to patients who had not been vaccinated (Median: 190.5*10^9/L) (P<0.05) **(B)**, higher eGFR (Median: 153.5ml/(min*1.73m2) compared to patients who had not been vaccinated (Median: 148.4ml/(min*1.73m2) (P<0.05) **(D)**, and higher albumin levels (Median: 38g/L) compared to patients who had not been vaccinated (Median: 35g/L) (P<0.05) **(F)**, Patients who had received three doses of vaccine had lower creatinine levels (Median: 44umol/L) compared to patients who had not been vaccinated (Median: 45.4umol/L) (P<0.05) **(C)**, and lower aspartate aminotransferase levels (Median: 17U/L) compared to patients who had not been vaccinated (Median: 19U/L) (P<0.05) **(E)**. * P<0.05, **P<0.01, ***P<0.001, and ***P<0.0001.

At admission, patients who had received three doses of vaccine had higher lymphocyte (Median: 1.575*10^9/L) compared to patients who had not been vaccinated (Median: 1.300*10^9/L) (P<0.05) (**Figure 2A**), higher platelet counts (Median: 210.5*10^9/L) compared to patients who had not been vaccinated (Median: 190.5*10^9/L) (P<0.05) (**Figure 2B**), higher eGFR (Median: 153.5ml/(min*1.73m^2^) compared to patients who had not been vaccinated (Median: 148.4ml/(min*1.73m^2^) (P<0.05) (**Figure 2D**), and higher albumin levels (Median: 38g/L) compared to patients who had not been vaccinated (Median: 35g/L) (P<0.05) (**Figure 2F**), Patients who had received three doses of vaccine had lower creatinine levels (Median: 44umol/L) compared to patients who had not been vaccinated (Median: 45.4umol/L) (P<0.05) (**Figure 2C**), and lower aspartate aminotransferase levels (Median: 17U/L) compared to patients who had not been vaccinated (Median: 19U/L) (P<0.05) (**Figure 2E**), However, there were no statistically significant differences in the levels of IL-6, IL-8, interferon alpha, interferon gamma, CD4+T lymphocytes, CD8+ T lymphocytes, D-dimer, or fibrin degradation products between the vaccinated and unvaccinated groups (P>0.05). (**Figure 2**).

**Figure 3:**
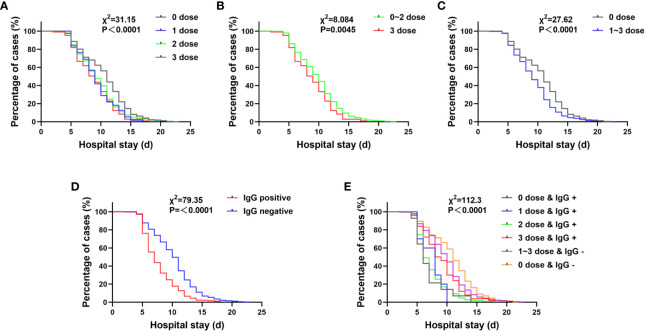
The influence of various factors on the hospitalization time of pregnant women with COVID-19. **(A–C)** The hospitalization time of pregnant women after vaccination is shortened. 0 dose vs 3 doses: χ^2 = ^21.99, *P*< 0.0001; 0 dose vs 2 doses: χ^2 = ^20.76, *P*< 0.0001; 0 dose vs 1 dose: χ^2 = ^9.83, *P* = 0.0017. **(D)** The length of hospitalization for pregnant women with SARS-CoV-2 IgG positive at admission was significantly shortened.(*P*< 0.0500). **(E)** (a) When reinfected, individuals previously infected with SARS-CoV-2 (0 dose & IgG +) had the shortest hospitalization time. 0 dose & IgG + vs 3 doses & IgG +: χ^2 = ^4.650, *P* = 0.0311; 0 dose & IgG + vs 0 dose & IgG -: χ^2 = ^20.03, *P*< 0.0001; 0 dose & IgG + vs 1~3 doses & IgG -: χ^2 = ^12.65, *P* = 0.0004; (b) Even if only one dose of vaccine is administered, as long as the SARS-CoV-2 IgG test is positive, the hospitalization time is shorter than that of patients who received three doses of vaccine but have a negative IgG response. 1 dose & IgG + vs 1~3 doses & IgG -: χ^2 = ^9.865, *P* = 0.0017; 2 doses & IgG + vs 1~3 doses & IgG -: χ^2 = ^56.2, *P*< 0.0001; (c) Although the IgG test result after vaccination was negative, the hospitalization time of those who were vaccinated and had a negative IgG test result was shorter than that of those who were not vaccined and had a negative IgG test result. 1~3 doses & IgG – vs 0 dose & IgG -: χ^2 = ^15.06, *P*= 0.0001.

Patients who were positive for SARS-CoV-2 IgG or had been vaccinated at the time of admission had significantly shorter hospital stays, which was not related to vaccine dose (P<0.0001). (**Figure 3**).

Patients who were only vaccinated with one dose of vaccine and found to be SARS-CoV-2 IgG-positive at admission had a significantly shorter hospitalization time of 7.4 ± 2.0 days while patients who were vaccinated with three doses of vaccine but were SARS-CoV-2 IgG-negative had a hospitalization time of 9.9 ± 3.5 days (P = 0.0017). (**Figure 3**). This hospitalization time was still shorter than that of patients who were not vaccinated and were SARS-CoV-2 IgG-negative, which was 10.9 ± 3.8 days (P = 0.0001). Even without vaccination, patients who were SARS-CoV-2 IgG-positive at admission had the shortest hospitalization time of 6.9 ± 2.7 days. These patients may have been infected with SARS-CoV-2 in the past and have developed immunity against SARS-CoV-2, or the test results could be false positive. (**Figure 3**). A possibility can be abortive infection. Another, after infection these individuals might have developed immunity for reinfection (10–12).

### Other factors also influence the length of stay of pregnant women infected with COVID-19

**Figure 4:**
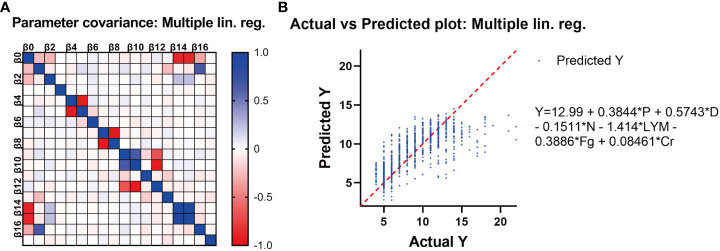
**(A)** The correlation between different variables. **(B)** Multivariate linear analysis of the potential factors affecting the length of stay of pregnant women with COVID-19. The length of hospitalization for pregnant women infected with SARS-CoV-2 Omicron variant is related to six factors, namely, Y: hospital stay, P: pregnancy stage, D: diagnostic type, N: Ct value (N gene), lym: lymphocyte count, FG: fibrinogen, and Cr: creatinine. Their relationship can be expressed as Y = 12.99 + 0.3844 * P + 0.5743 * D - 0.1511 * N - 1.414 * lym - 0.3886 * FG + 0.08461 * Cr.

In addition to vaccination, several other factors that may affect hospitalization time were analyzed through Spearman’s test, including pregnancy, pregnancy risk factors, diagnostic classification (asymptomatic = 0, mild = 1, moderate = 2), the interval between the last vaccination and hospitalization, SARS-CoV-2 specific IgG level, SARS-CoV-2 nucleic acid PCR Ct value, blood type, and common laboratory indicators. (**Table 3**). Important indicators with positive correlation were screened. Multilinear regression analysis showed that six factors were associated with hospitalization time. SARS-CoV-2 infection in late pregnancy was positively correlated with disease severity, creatinine level, and hospitalization time. It was found that the PCR Ct value of SARS-CoV-2 N gene, lymphocyte count, and fibrinogen level were negatively correlated with hospitalization time (P<0.050) (**Table 4**). In order to predict the hospitalization time of pregnant women with COVID-19-infection, we developed a model as follows: Y = 12.99 + 0.3844*P + 0.5743*D - 0.1511*N - 1.414*LYM - 0.3886*Fg + 0.08461*Cr, where Y represents hospitalization time, P represents pregnancy, D represents diagnostic classification, N represents SARS-CoV-2 CT value (N gene), LYM represents lymphocyte count, Fg represents fibrinogen, and Cr represents creatinine (**Figure 4**).

**Table 3:** Factors that may affect the length of stay of pregnant women infected with COVID-19.

Subject^#^	r	*P*
Age (years)	0.01885	0.5468
Pregnancy stage (early = 1, middle = 2, late = 3)	0.2068	< 0.0001
Pregnancy risk (purple + green = 1, purple + yellow = 2, purple + orange = 3)	0.09768	0.0018
Clinical manifestations (asymptomatic = 0, mild = 1, moderate = 2)	0.1443	< 0.0001
Vaccination (No = 0, Yes = 1)	-0.1443	< 0.0001
Doses of vaccine (0, 1, 2, 3)	-0.1531	< 0.0001
Days between last vaccination and on admission	0.06728	0.0738
SARS-CoV-2 IgG (negative = 0, positive = 1)	-0.2510	< 0.0001
Ct value of SARS-CoV-2 (N gene)	-0.6745	< 0.0001
Ct value of SARS-CoV-2 (ORF1ab)	-0.6665	< 0.0001
Blood type (A = 1, B = 2, AB = 3, O = 4)	-0.03115	0.3495
Lymphocyte count	-0.09631	0.0028
Neutrophil count	-0.1856	<0.0001
Platelet count	-0.1987	<0.0001
Leukocyte count	-0.1812	<0.0001
Fibrinogen	-0.1124	0.0005
D-dimer	0.01872	0.7011
eGFR	-0.2198	<0.0001
Creatinine	0.2102	<0.0001
Alanine aminotransferase	0.06036	0.0613
Aspartate aminotransferase	0.07520	0.0194
Albumin	-0.1075	0.0009
CD3-positive cells	-0.3997	0.0389
CD4-positive cells	-0.4713	0.0131
CD8-positive cells	-0.2903	0.1419

^#^The data were collected upon admission.

**Table 4:** Multivariate linear analysis of potential factors affecting the length of stay of pregnant women with COVID-19.

Multicollinearity	Variable^#^	Estimate	|t|	*P*
**β0**	Intercept	12.99	3.914	0.0001
**β1**	Pregnancy stage (early = 1, middle = 2, late = 3)	0.3844	2.255	0.0245
**β2**	Clinical manifestations (asymptomatic = 0, mild = 1, moderate = 2)	0.5743	2.125	0.0339
**β3**	Pregnancy risk (purple + green = 1, purple + yellow = 2, purple + orange = 3)	0.09581	0.6736	0.5008
**β4**	Vaccination (No = 0, Yes = 1)	-0.5868	1.071	0.2846
**β5**	Doses of vaccine (0, 1, 2, 3)	0.04397	0.1753	0.8609
**β6**	SARS-CoV-2 IgG (negative = 0, positive = 1)	-0.1003	0.3843	0.7009
**β7**	Ct value of SARS-CoV-2 (N gene)	-0.1511	3.172	0.0016
**β8**	Ct value of SARS-CoV-2 (ORF1ab)	-0.0684	1.478	0.1400
**β9**	Lymphocyte count	-1.414	5.402	<0.0001
**β10**	Neutrophil count	-0.2412	1.534	0.1255
**β11**	Platelet count	-0.000651	0.3477	0.7282
**β12**	Leukocyte count	0.1908	1.231	0.2188
**β13**	Fibrinogen	-0.3886	2.959	0.0032
**β14**	eGFR	0.0133	1.445	0.1488
**β15**	Creatinine	0.08461	2.322	0.0206
**β16**	Albumin	-0.01196	0.3623	0.7173
**β17**	Aspartate aminotransferase	-0.0001907	0.05474	0.9564

^#^The data were collected upon admission. Pregnancy stage (early = 1, middle = 2, late = 3); Clinical manifestations (asymptomatic = 0, mild = 1, moderate = 2); Pregnancy risk (purple + green = 1, purple + yellow = 2, purple + orange = 3); Vaccination (No = 0, Yes = 1); Doses of vaccine (0, 1, 2, 3); SARS-CoV-2 IgG (negative = 0, positive = 1).

### Effect of vaccination on newborns born to mothers with COVID-19

A total of 118 COVID-19 patients gave birth to 120 newborns during hospitalization. Among them, 76 patients were vaccinated with inactivated COVID-19 vaccines, and 42 patients were not vaccinated. (**Table 5**). The vaccination status before infection with SARS-CoV-2 did not affect the mode of delivery, pregnancy outcomes (premature delivery, premature rupture of membranes (P-PROM), full-term delivery (term birth), neonatal malformation, neonatal death), amniotic fluid contamination index, body weight and length, malformation rate, or Apgar score (P>0.05). (**Table 5**).

**Table 5:** Clinical characteristics of newborns born to mothers with COVID-19.

Subject (case, %)	Total (n = 120)	Doses of vaccine (pregnant women)		*P*
		(A) 0 dose (n = 44)	(B) 1~3 dose (n = 76)	
Gender^#^				>0.9999
Male	64 (53.3)	23 (52.3)	41 (53.9)	
Female	56 (46.7)	21 (47.7)	35 (46.1)	
Mode of delivery^#^				0.5667
Natural	52 (43.3)	21 (47.7)	31 (40.8)	
C-section	68 (56.7)	23 (52.3)	45 (59.2)	
Pregnancy outcome^#^				
Premature	6 (5)	3 (6.8)	3 (3.9)	0.6677
P-PROM	7 (5.8)	3 (6.8)	4 (5.3)	0.7062
Term birth	107 (89.2)	38 (86.4)	69 (90.8)	0.5454
Neonatal malformation	4 (3.3)	1 (2.3)	3 (3.9)	>0.9999
Neonatal death	0 (0)	0 (0)	0 (0)	>0.9999
Amniotic fluid^#^				
Clean	109 (90.8)	39 (88.6)	70 (92.1)	0.5292
I°polluted	5 (4.2)	3 (6.8)	2 (2.6)	0.3548
II°polluted	1 (0.8)	0 (0)	1 (1.3)	>0.9999
III°polluted	5 (4.2)	2 (4.5)	3 (3.9)	>0.9999
**Positive SARS-CoV-2 IgG^#^**	0 (0)	0 (0)	0 (0)	>0.9999
**Positive SARS-CoV-2 IgM^#^**	0 (0)	0 (0)	0 (0)	>0.9999
**Positive nucleic acid^#^**	0 (0)	0 (0)	0 (0)	>0.9999
**Positive nucleic acid**^a^	0 (0)	0 (0)	0 (0)	>0.9999
**Weight (g)^#^**	3221 ± 471	3211 ± 510	3227 ± 450	0.7377
Height (cm)^#^	49.7 ± 1.0	49.7 ± 1.0	49.7 ± 1.0	0.5238
Apgar 1-min score^#^	9.73 ± 0.69	9.84 ± 0.37	9.67 ± 0.82	0.3964
Apgar 5-min score^#^	9.95 ± 0.31	10.0 ± 0	9.92 ± 0.39	0.2952
Apgar 10-min score^#^	9.96 ± 0.30	10.0 ± 0	9.93 ± 0.38	0.2975

^#^The data were collected during delivery.

a The data were collected one week after birth.

Among the 76 vaccinated patients, 68 were vaccinated before pregnancy, and 8 were vaccinated during pregnancy. (**Table 6**). The comparison between the two groups showed that the vaccination time did not affect the delivery mode, pregnancy outcome, amniotic fluid pollution level, body weight and length, malformation rate, and Apgar score (P>0.05). (**Table 6**).

**Table 6:** The influence of the mother’s first vaccination time on the newborn.

Subject (case, %)^#^	Total (n = 76)	Timing of 1st vaccination (pregnant women)		*P*
		(A) before pregnancy (n = 68)	(B) after pregnancy (n = 8)	
Gender				0.7188
Male	41 (53.9)	36 (52.9)	5 (62.5)	
Female	35 (46.1)	32 (47.1)	3 (37.5)	
Mode of delivery				>0.9999
Natural	31 (40.8)	28 (41.2)	3 (37.5)	
C-section	45 (59.2)	40 (58.8)	5 (62.5)	
Pregnancy outcome				
Premature	3 (3.9)	3 (4.4)	0 (0)	>0.9999
P-PROM	4 (5.3)	2 (2.9)	2 (25)	0.0527
Term birth	69 (90.8)	63 (92.6)	6 (75)	0.1560
Neonatal malformation	3 (3.9)	3 (4.4)	0 (0)	>0.9999
Neonatal death	0 (0)	0 (0)	0 (0)	>0.9999
Amniotic fluid				
clean	70 (92.1)	62 (91.2)	8 (100)	>0.9999
I°polluted	2 (2.6)	2 (2.9)	0 (0)	>0.9999
II°polluted	1 (1.3)	1 (1.5)	0 (0)	>0.9999
III°polluted	3 (3.9)	3 (4.4)	0 (0)	>0.9999

^#^The data were collected during delivery.

To verify the possibility of vertical transmission of SARS-CoV-2, we tested 120 newborns for SARS-CoV-2 nucleic acid and SARS-CoV-2-specific IgG and IgM levels, and the results were all negative. (**Table 5**). All the newborns lived with their mothers and were breastfed. One week after birth, the nucleic acid test results for SARS-CoV-2 in the newborns were all negative. (**Table 5**).

## Discussion

A recent study of nearly 1 million cases confirmed the safety of COVID-19 inactivated vaccines in China, although mild adverse reactions such as transient low fever, headache or fatigue were observed (13–15). These vaccines are theoretically safe for women who are preparing pregnancy or are pregnant. In 2021, the US Center for Disease Control and Prevention (CDC) suggested that any authorized COVID-19 vaccine can be used for pregnant or lactating women (16). In addition, the American College of Obstetrics and Gynecology and SMFM strongly recommend vaccination for pregnant and breastfeeding women. There are no restrictions on the type or dose of vaccine during pregnancy (17). However, due to the lack of maternal vaccination data in China, the impact of COVID-19 vaccine on pregnant women, fetuses, and newborns is still uncertain. In addition, China has not yet implemented large-scale vaccination for pregnant women.

Previous studies have confirmed that pregnancy increases the risk of serious illness associated with COVID-19. However, it is still unclear which specific pregnant women are more likely to be infected with SARS-CoV-2. In this study, we analyzed data of 1,024 pregnant women diagnosed with COVID-19. Our findings suggest that most patients were in the third trimester of pregnancy, which may be due to increased diaphragmatic muscle elevation, increased oxygen consumption, and impaired immune functions during this stage. The low level of SARS-CoV-2 IgG further confirms this evidence; Only 10.3% of late pregnancy population tested positive for SARS-CoV-2 IgG, which significantly lower compared to early pregnancy population (28.8%).

Pregnant women with COVID-19 are more likely to have mild symptoms on admission. However, only a small number of patients have developed symptoms such as fever, cough, shortness of breath, vomiting, diarrhea, and rash. The low toxicity of the Omicron strain may explain this. In addition, our study found that the status of vaccination or the number of vaccinations does not predict the clinical symptoms or severity of disease in pregnant women admitted to the hospital. Among pregnant women who had received three doses of vaccine, 49.5% were positive for SARS-CoV-2 IgG, significantly higher than 4.7% of those who had not received vaccine, 19.2% of those who had received one dose of vaccine, and 20.3% of those who had received two doses of vaccine. Such a positive correlation between SAR-COV-2 IgG positivity and the number of vaccinations were in line with what had been observed in general populations, strengthening the notion that more vaccinations with appropriate intervals could cultivate a more robust antibody memory response with higher titers and longer durations (10, 12, 18, 19). Based on our data, we conclude that, for pregnant women, multiple vaccinations during pregnancy or preparation for pregnancy conferred beneficial effects upon the later CoVID-19 infections, with the degree of protection positively correlated with the vaccine doses. It should be noted that this conclusion was drawn from comparing the effects of one, two, and three doses of vaccines. Whether more vaccinations beyond three doses could further enhance protection is an open question awaiting future investigation.

In addition, compared with patients who did not receive vaccination, patients who received three doses of the vaccine showed higher lymphocyte and platelet counts, as well as higher eGFR and albumin levels, while also exhibiting lower creatinine and aspartate aminotransferase levels. Vaccination may provide protection and reduce the severity of maternal disease when infected with SARS-CoV-2. The hospitalization time of pregnant women who received three doses of vaccine was only 8.8 ± 3.3 days, which was significantly shorter than that of pregnant women who did not receive the vaccine (11.0 ± 3.9 days). This finding shows that repeated vaccination of COVID-19 vaccine can speed up virus clearance and shorten the duration of disease in pregnant women. In **Figure 2A**, only three doses of vaccination significantly increased the lymphocyte count in the blood of pregnant women. We believe that this may be consistent with the positive SARS-CoV-2 specific IgG, and is also due to the more significant stimulation of pregnant women’s immune cells by more vaccinations, leading to immune cell proliferation and antibody secretion. However, as no pregnant women have received 4 or more doses of vaccine, it is unclear whether receiving 4 doses is better than receiving 3 doses. According to current data, the higher the number of vaccinations, the higher the antibody positive rate and the lymphocyte count.

Most importantly, even if only one dose of vaccine is administered, as long as the individual is positive for SARS-CoV-2 IgG, their hospitalization time (7.4 ± 2.0 days) is still shorter than that of individuals who have received three doses of vaccine but are negative for SARS-CoV-2 IgG (9.9 ± 3.5 days), with a statistically significant difference (P = 0.0017). Even if the IgG test is negative after vaccination, the hospitalization time (9.9 ± 3.5 days) is still shorter than that of individuals who have not been and who are negative for antibodies (10.9 ± 3.8 days), again with a statistically significant difference (P = 0.0001). These observations suggest that the three-dose vaccination regimen is more effective, and for those who hesitant about whether to receive multiple doses of vaccine, IgG testing was after vaccination is necessary.

Our study also showed that infection with SARS-CoV-2 in the third trimester of pregnancy and high viral nucleic acid PCR Ct values were positively correlated with disease severity, while high lymphocyte counts were negatively correlated with disease severity and length of hospital stay. These observations can help identify pregnant women with high-risk factors early and focus on preventing disease progression.

Regarding the safety of vaccines, previous studies conducted in other countries have shown that pregnant women experience slightly higher levels of pain at the injection site compared to non-pregnant women, and have slightly lower incidences of headache, muscle ache, chills, and fever. Among the 827 pregnant women in the Vsafe Pregnancy Registry, the incidence of adverse pregnancy and neonatal outcomes, such as miscarriage, premature delivery, small gestational age, and congenital anomalies, is comparable to published background rates. In addition, there were no reports of newborn deaths (20). Another retrospective cohort study has also found that vaccination of pregnant women was not associated with serious adverse events within 42 days (21). It is worth noting that rare adverse reactions, such as thrombocytopenia syndrome (TTS), have also been reported after vaccination with adenovirus vaccines (22, 23). For safety reasons, the United Kingdom prefers to use mRNA vaccines rather than adenovirus vaccines (24, 25). In our study, the only vaccine used for domestic vaccination is the inactivated COVID-19 vaccine. These vaccines retain the immunogenic components that trigger the immune response in the human body, while losing their infectivity and replication ability, thus ensuring safety and effectiveness.

A total of 118 patients gave birth to 120 newborns during their hospital stay. Our findings suggest that maternal vaccination is safe for newborns, as it does not affect delivery mode, pregnancy outcome, weight and length, malformation rate, or Apgar score, regardless of whether vaccination was given before or during pregnancy.

Another key question is whether COVID-19 will cross the placenta and directly harm the fetus. It is crucial to pay attention to this during pregnancy, childbirth, and breastfeeding. We tested 120 newborns for SARS-CoV-2 nucleic acid, IgG, and IgM levels, and found that all of them were negative, thus eliminating the possibility of vertical transmission. This is consistent with previous studies conducted in other countries, indicating that the prevalence of vertical transmission is low. This may be related to the low level of SARS-CoV-2 and the reduced co-expression of angiotensin-converting enzyme 2 (ACE2) and transmembrane serine protease 2 (TMPRSS2) required for entry into placental cells (26, 27). The pregnant women in this study lived with breastfed newborns. No positive nucleic acid tests was detected in the newborn after one week of birth, indicating that maternal with the newborn is safe under the continuous use of surgical masks, hand hygiene, and breast cleaning. (**Supplementary Figure S1**). Many foreign guidelines also advocate allowing newborns to adapt to infected mothers, especially if the mother is asymptomatic (28, 29).

Our strategies for preventing neonatal infection with SARS-CoV-2 includes minimizing labor processes, avoiding invasive surgery during delivery, reducing the contact time between the newborn and the maternal birth canal, wiping away mucus and amniotic fluid as soon as possible after birth, and promptly transferring the newborn from the operating room or delivery room. A study conducted in the United States on nearly 99,000 pregnant women infected with SARS-CoV-2 found that 109 patients died, with a mortality rate of 0.1%. On the other hand, our study did not record any deaths among the 1,024 pregnant women, which may be due to our screening and timely intervention for high-risk patients (30).

## Data availability statement

The raw data supporting the conclusions of this article will be made available by the authors, without undue reservation.

## Ethics statement

The studies involving humans were approved by the Ethics Committee of the Shanghai Public Health Clinical Center (2022-S067-01). The studies were conducted in accordance with the local legislation and institutional requirements. The participants provided their written informed consent to participate in this study.

## Author contributions

HD: Writing – original draft. YJ: Writing – original draft. MS: Writing – original draft. ML: Writing – review & editing. JS: Writing – review & editing. WQ: Writing – review & editing. GZ: Writing – review & editing. YXL: Writing – review & editing. TL: Writing – review & editing. YL: Writing – review & editing. XF: Writing – review & editing.

## Supplementary material

Supplementary Figure 1 Shanghai’s experience in preventing vertical transmission of COVID-19.

Supplementary Table 1 Clinical characteristics of COVID-19 infection in different stages of pregnancy.
