# Supplementary material for: Safety and efficacy of COVID-19 vaccine immunization during pregnancy in 1024 pregnant women infected with the SARS-CoV-2 Omicron virus in Shanghai, China
Source: Front Immunol. 2024 Jan 16;14:1303058. doi: 10.3389/fimmu.2023.1303058 (PMC10826606; doi:10.3389/fimmu.2023.1303058)
Supplement: Supplementary Figure 1 — Shanghai’s experience in preventing vertical transmission of COVID-19. [file DataSheet_1.docx]

**Supplementary**

**Figure S1: Shanghai's experience in preventing vertical transmission of COVID-19.**

**
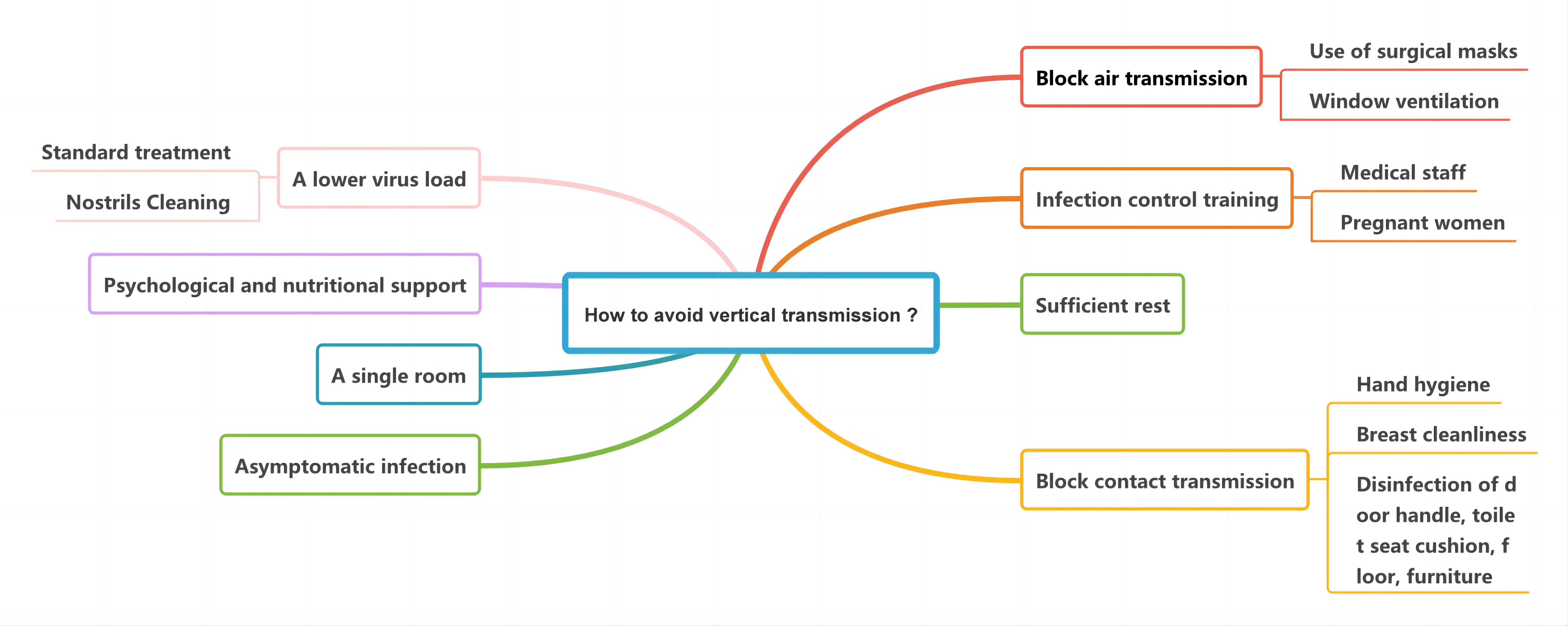
**

**Supplementary**

**Table S1. Clinical characteristics of COVID-19 infection in different stages of pregnancy.**

| **Subject (case, %)** | **Total (n=1024)** | **Pregnancy stages** | | | **Statistical value** | ***P*** |
| --- | --- | --- | --- | --- | --- | --- |
|  |  | 1. **Early (n = 282)** | **(B) Middle (n = 276)** | **(C) Late (n = 466)** |  |  |
| **Age (years)** |  |  |  |  | χ^2^ = 0.9873 | 0.6104 |
| **18–34** | 849 (82.9) | 233 (82.6) | 234 (84.8) | 382 (82.0) |  |  |
| **≥35** | 175 (17.1) | 49 (17.4) | 42 (15.2) | 84 (18.0) |  |  |
| **Pregnancy risk^a^** | |  |  |  | χ^2^ = 22.49 | 0.0002 |
| Purple + green | 535 (52.2) | 171 (60.6) | 156 (56.5) | 208 (44.6) | χ^2^ = 20.8 | <0.0001 |
| Purple + yellow | 379 (37.0) | 91 (32.3) | 93 (33.7) | 195 (41.8) | χ^2^ = 8.693 | 0.0130 |
| Purple + orange | 110 (10.7) | 20 (7.1) | 27 (9.8) | 63 (13.5) | χ^2^ = 7.932 | 0.0190 |
| **Clinical manifestations^b^** | | |  |  | χ^2^ = 2.677 | 0.6132 |
| Asymptomatic | 865 (84.5) | 236 (83.7) | 235 (85.1) | 394 (84.5) | χ^2^ = 0.2296 | 0.8916 |
| Mild | 157 (15.3) | 46 (16.3) | 41 (14.9) | 70 (15.0) | χ^2^ = 0.2916 | 0.8643 |
| Moderate | 2 (0.2) | 0 (0) | 0 (0) | 2 (0.5) |  |  |
| **Clinical symptoms^#^** | |  |  |  | χ^2^ = 7.063 | 0.5298 |
| Fever | 38 (3.7) | 15 (5.3) | 9 (3.3) | 14 (3.0) | χ^2^ = 6.605 | 0.0482 |
| Coughing/difficulty breathing | 62 (6.1) | 17 (6.0) | 14 (5.1) | 31 (6.7) | χ^2^ = 0.7611 | 0.6835 |
| Vomiting | 8 (0.8) | 5 (1.8) | 0 (0) | 3 (0.6) |  |  |
| Diarrhea | 2 (0.2) | 1 (0.4) | 0 (0) | 1 (0.2) |  |  |
| Rashes | 5 (4.9) | 1 (0.4) | 1 (0.4) | 3 (0.6) |  |  |
| **Image changes^#^** | 0 (0) | 0 (0) | 0 (0) | 2 (4.3) |  |  |
| **Positive SARS-CoV-2 IgG^#^** | 180 (18.4) | 77 (28.8) | 57 (21.7) | 46 (10.3) | χ^2^ = 40.6 | < 0.0001 |
| **Positive SARS-CoV-2 IgM^#^** | 5 (0.5) | 5 (1.9) | 0 (0) | 0 (0) |  |  |
| **Hospital stay (days)** | 9.8 ± 3.8 | 8.7 ± 3.3 | 9.5 ± 3.3 | 11 ± 4.3 | A vs. B | 0.0082 |
|  |  |  |  |  | A vs. C | < 0.0001 |
|  |  |  |  |  | B vs. C | 0.0004 |

^a^ The National Health and Family Planning Commission issued a pregnancy risk assessment form for women, which was divided into five colors based on the severity of the risk: green (low risk), yellow (general risk), orange (high-risk), red (extremely high-risk), and purple (infectious disease).

^b^ The definition of "mild" was stated as mild clinical symptoms without any alterations in the computed tomography (CT) images; The definition of "moderate" was assigned as mild clinical symptoms accompanied by alterations in CT images.

^#^ The data collected on admission.
